# Focused attention vs. crossmodal signals paradigm: deriving predictions from the time-window-of-integration model

**DOI:** 10.3389/fnint.2012.00062

**Published:** 2012-08-29

**Authors:** Hans Colonius, Adele Diederich

**Affiliations:** ^1^Department of Psychology, Carl von Ossietzky Universitaet OldenburgOldenburg, Germany; ^2^School of Humanities and Social Sciences, Jacobs University BremenBremen, Germany

**Keywords:** focused attention, cross-modal, time-window-of-integration, Bayesian decision theory, exponential distribution

## Abstract

In the crossmodal signals paradigm (CSP) participants are instructed to respond to a set of stimuli from different modalities, presented more or less simultaneously, as soon as a stimulus from any modality has been detected. In the focused attention paradigm (FAP), on the other hand, responses should only be made to a stimulus from a pre-defined target modality and stimuli from non-target modalities should be ignored. Whichever paradigm is being applied, a typical result is that responses tend to be faster to crossmodal stimuli than to unimodal stimuli, a phenomenon often referred to as “crossmodal interaction.” Here, we investigate predictions of the time-window-of-integration (TWIN) modeling framework previously proposed by the authors. It is shown that TWIN makes specific qualitative and quantitative predictions on how the two paradigms differ with respect to the probability of multisensory integration and the amount of response enhancement, including the effect of stimulus intensity (“inverse effectiveness”). Introducing a decision-theoretic framework for TWIN further allows comparing the two paradigms with respect to the predicted optimal time window size and its dependence on the prior probability that the crossmodal stimulus information refers to the same event. In order to test these predictions, experimental studies that systematically compare crossmodal effects under stimulus conditions that are identical except for the CSP-FAP instruction should be performed in the future.

## 1. Introduction

In the crossmodal signals paradigm^1^ (CSP) participants are instructed to respond to a set of stimuli from different modalities, presented more or less simultaneously, as soon as a stimulus from any modality has been detected. In the focused attention paradigm (FAP), on the other hand, responses should only be made to a stimulus from a pre-defined target modality and stimuli from non-target modalities should be ignored. Thus in FAP, but not in CSP, participants are required to distinguish between target and non-target modality. Whichever paradigm is being applied, a typical result is that responses tend to be faster to crossmodal stimuli than to unimodal stimuli, a phenomenon often referred to as “intersensory (or crossmodal) interaction,” already reported in Todd (1912). Many attempts have been made on both the behavioral and neurophysiological level to understand the dynamics of mechanisms that underlie these crossmodal effects (cf. Stein, 2012, for a recent overview). Up to now, however, reaction time models have predominantly been concerned with CSP. The purpose of this paper is to demonstrate how both types of paradigm can be accounted for within the time-window-of-integration (TWIN) modeling framework proposed by the authors (Colonius and Diederich, 2004; Diederich and Colonius, 2004). Moreover, we will extend the decision-making framework for TWIN to include both CSP and FAP. Under appropriate empirical restrictions, TWIN predicts crossmodal interaction effects in one of the paradigms (CSP, say) given crossmodal interaction effects observed in the other (FAP). While permitting a stringent test of this modeling framework by comparing the implementation of CSP and FAP in TWIN, we moreover strive to get a deeper understanding of the cognitive processes elicited by these two different crossmodal paradigms.

The classic explanation for a speed-up of responses to crossmodal stimuli in CSP has been that subjects start preparing a response as soon as the first stimulus has been detected (Raab, 1962). Taking detection times to be random variables and adding some technical assumptions, observed reaction time is represented as the minimum of the reaction times to, say, visual and auditory signals leading to a purely statistical facilitation effect (probability summation) in response speed. Numerous studies have shown that this *separate activation* or *race model* is not sufficient to explain the observed speedup in reaction time, however, (see Diederich and Colonius, 2004, for a review). Using the race model inequality (RMI) (Miller, 1982; Colonius and Diederich, 2006) as a benchmark test, responses to bimodal stimuli have been found to be faster than predicted by statistical facilitation, in particular, when the stimuli were spatially aligned. Although the RMI test has sometimes been applied to data from both types of paradigm, its validity for FAP data seems problematic as long as no specific assumptions about the effect of a stimulus from the non-target modality winning the race are being made. Moreover, the race model gives no explanation for the decrease in facilitation observed with variations in many crossmodal stimulus properties, e.g., increasing spatial disparity between the stimuli.

An alternative model type *coactivation models* assumes that activation, raised in different sensory channels by presenting crossmodal stimuli, is combined to satisfy a single criterion for response initiation (Miller, 1982). Coactivation models predict faster average reaction time to multiple stimuli compared to single stimuli because the combined activation reaches that criterion faster. Mathematical instantiations of this model type include *superposition* or *counter models* (Schwarz, 1989; Diederich and Colonius, 1991; Diederich, 1995) and *diffusion models* (Schwarz, 1994; Diederich, 1995). Although these models have been quite successful in describing various empirical data sets for CSP, they have as yet no provision to deal with FAP. Note that neither coactivation nor race models can predict inhibition, i.e., sometimes responses to crossmodal stimuli are slower than to unimodal ones.

## 2. Time window of integration modeling framework: general description

The *time-window hypothesis* holds that information from different sensory modalities must not be presented too far apart in time so that integration into a multisensory perceptual unit may occur. The concept, already mentioned over 20 years ago (Meredith et al., 1987; Stein and Meredith, 1993), recently enjoyed increasing popularity on both the neural and behavioral levels of observation (e.g., Lewald et al., 2001; Meredith, 2002; Lewald and Guski, 2003; Spence and Squire, 2003; Wallace et al., 2004; Bell et al., 2005, 2006; Navarra et al., 2005; Romei et al., 2007; Rowland and Stein, 2007; Rowland et al., 2007; Van Wassenhove et al., 2007; Musacchia and Schroeder, 2009; Powers et al., 2009; Royal et al., 2009). Although a “window of integration” has previously been defined for both spatial and temporal aspects of a crossmodal experiment (e.g., Wallace et al., 2004) and has even been suggested for higher-level aspects like semantic congruity (e.g., Van Atteveldt et al., 2007), we will confine discussion to the temporal dimension within the reaction time context considered here. To the best of our knowledge, however, the TWIN model framework) is the only effort to develop an explicit quantitative rendering of a crossmodal time-window mechanism (Colonius and Diederich, 2004, 2012) and to introduce a decision-theoretic perspective on predicting an optimal time window (Colonius and Diederich, 2011).

Given that the basic concept of a “race” among neural activities elicited in separate peripheral sensory pathways, i.e., at a very early stage of processing, has considerable intuitive plausibility, the TWIN model retains this concept which is central to separate activation models. The first stage is complemented by a subsequent compound stage of converging processes which comprise neural integration of the input and preparation of a response. This second stage is defined by default: it includes all later, possibly temporally overlapping, processes that are not part of the peripheral processes in the first stage.

The central assumption of the model concerns the temporal configuration needed for multisensory integration to occur:

[**TWIN assumption**] *Multisensory integration occurs only if all peripheral processes of the first stage terminate within a given temporal interval, the “time window of integration.”*

Thus, the window acts as a “filter” determining whether or not afferent information delivered from different sensory organs is registered close enough in time to trigger multisensory integration. Passing the filter is necessary but not sufficient for crossmodal interaction to occur since the amount of interaction may also depend on several other aspects of the stimulus setting, like spatial configuration of the stimuli. The *amount* of crossmodal interaction manifests itself in an increase or decrease of second stage processing time but it is assumed not to depend on how far apart in time the stimuli have been presented (stimulus onset asynchrony, SOA).

For FAP, the TWIN assumption is further constrained in one important respect:

[**FAP condition**] *Crossmodal interaction in FAP only occurs if (i) a non-target stimulus wins the race in the first stage opening the time window of integration, such that (ii) the termination of the target peripheral process falls into the window.*

One interpretation is that a winning non-target will keep the system in a state of heightened reactivity such that the upcoming target stimulus, if it falls into the time window, will trigger crossmodal interaction. For saccadic eye movements, for example, this may correspond to a gradual inhibition of fixation neurons (in *superior colliculus*) and/or *omnipause* neurons (in *midline pontine* brain stem). If a stimulus from the target modality is the winner of the race in the peripheral channels, second stage processing is initiated without any multisensory integration mechanism being involved.

Although these TWIN model assumptions clearly oversimplify matters, the framework generates several experimentally testable predictions, some of which have already found empirical support in recent studies (cf. Diederich and Colonius, 2007a,b, 2008a,b). Since physically identical stimuli can be presented in both FAP and CSP under the same spatiotemporal configuration, any systematic differences observed in the corresponding reaction times have to be due to the instructions being different. Thus, differences between the two paradigms may allow one to assess, and possibly separate from one another, the contribution of top-down processes and bottom-up processes in multisensory integration.

## 3. The formal presentation of TWIN for FAP and CSP

For the crossmodal condition, the race in the first stage is based on postulating statistically independent, non-negative continuous random variables representing the durations of the peripheral processes. With *V* and *A* denoting these visual and auditory processing times^2^, respectively, the central TWIN assumption introduced above translates into

(1) |V−A|<ω,

i.e., peripheral processes *V* and *A* terminate within an integration window of width ω. This inequality is the condition for the *event of integration* to occur in the case of CSP, denoted *I*_CSP_, and it is obviously equivalent to the union of the events

$$\{V<A<V+\omega \}\cup \{A<V<A+\omega \}\equiv I_{\text{CSP}}.$$

For the FAP with, say, the visual as target modality, the condition for integration is, by translating the FAP condition stated above,

$$I_{\text{FAP}}=\{A<V<A+\omega \}.$$

Therefore, under identical stimulus conditions,

$$I_{\text{FAP}}\equiv I_{\text{CSP}}\cap \{A\text{ is the winner of the race}\}.$$

It follows that any realization of the peripheral processing times *V* and *A* that leads to an opening of the time window under the focused attention instruction also leads to that event under the crossmodal signals instruction, i.e., *I*_FAP_⊂ I_CSP_. Thus, the probability of integration under crossmodal signals instruction can not be smaller than that under focused attention instruction: Pr(*I*_FAP_) ≤ Pr(*I*_CSP_), given identical stimulus conditions hold.

### 3.1. Expected crossmodal reaction time for FAP and CSP

Although events *I*_FAP_ and *I*_CSP_ are not empirically observable, the numerical ordering of their associated probabilities leads to a corresponding prediction about mean crossmodal reaction times. Indeed, according to the two-stage assumption, total reaction time in the crossmodal condition can be written as a sum of two random variables:

(2) $${\text{RT}}_{VA}=W_{1}+W_{2},$$

where *W*_1_ and *W*_2_ refer to first and second stage processing times, respectively. With Pr(*I*) the probability that integration occurs in CSP or FAP, expected saccadic reaction time in the crossmodal condition (E[*RT*_*VA*_]) then is:

$$\begin{array}{l} \text{E}[RT_{VA}]=\text{E}[W_{1}]+\text{E}[W_{2}]\\ \;=\text{E}[W_{1}]+\text{Pr}[I]\text{E}[W_{2}|I]+(1-\text{Pr}[I])\text{E}[W_{2}|\text{not-}I]\\ \;=\text{E}[W_{1}]+\text{E}[W_{2}|\text{not-}I]-\text{Pr}[I](\text{E}[W_{2}|\text{not-}I]-\text{E}[W_{2}|I]), \end{array}$$

where E[W_2_|*I*] and E[W_2_|not-*I*] denote the expected second stage processing time conditioned on interaction occurring (*I*) or not occurring (not-*I*), respectively. Putting

$$\Delta \equiv \text{E}[W_{2}|\text{not-}I]-\text{E}[W_{2}|I],$$

this becomes

(3) $$\text{E}[RT_{VA}]=\text{E}[W_{1}]+\text{E}[W_{2}|\text{not-}I]-\text{Pr}[I]\;\cdot \;\Delta .$$

The term Pr[*I*] · Δ can be interpreted as a measure of the expected saccadic RT interaction effect in the second stage with positive Δ values corresponding to facilitation, negative ones to inhibition. The duration of the first stage, *W*_1_, must be defined differently for CSP and FAP:

(4) $$W_{1}=\left\{ \begin{array}{l} \min(V,\text{ A) for CSP,}\\ V\text{ for FAP,} \end{array}\right.$$

assuming the visual as target modality in FAP. Thus, for the expected overall reaction time in the crossmodal condition

(5) $$\text{E}[RT_{VA}]=\{\begin{array}{ll} \text{E}[\min(V,A)]+\mu -P(I_{\text{CSP}})\cdot \Delta , & \text{for CSP},\\ \text{E}[V]+\mu -P(I_{\text{FAP}})\cdot \Delta , & \text{for FAP,} \end{array}$$

with μ≡ E[*W*_2_|not-*I*].

The last equation allows to predict how (observable) mean reaction times for FAP and CSP may differ. In fact, under identical stimulus conditions and assuming facilitation occurs (i.e., Δ >0), expected crossmodal reaction time can never be longer in CSP than in FAP because both E[min(*V*, *A*)]≤ E[*V*] and Pr(*I*_FAP_) ≤ Pr(*I*_CSP_). Thus,

$$\text{E}[RT_{VA}|\text{CSP}]\leq \text{E}[RT_{VA}|\text{FAP}].$$

Some empirical support for this prediction was found in an unpublished experiment from our lab, but further empirical testing is required.

### 3.2. Crossmodal response enhancement for FAP and CSP

In the unimodal condition, no interaction is possible. Thus,

(6) $$\text{E}[RT_{\text{unimodal}}]=\text{E}[W_{1}]+\text{E}[W_{2}|\text{not-}I].$$

Note that in order to relate processing durations in the unimodal conditions to those occurring in the crossmodal conditions, one has to introduce a basic assumption, known as “context independence” or “context invariance” (cf. Ashby and Townsend, 1986; Luce, 1986; Colonius, 1990; Townsend and Eidels, 2011). Informally, it amounts to assuming that the (marginal) distributions of random variables (like *V* and *A*) occurring in the crossmodal conditions are identical to the distributions of the corresponding random variables occurring in the unimodal conditions. Although not empirically testable, context invariance has been widely accepted as a plausible modeling constraint and will be used here as well.

In analogy to measuring multisensory enhancement in neural responses (cf. Meredith and Stein, 1986; Anastasio et al., 2000), the amount of crossmodal reaction time interaction is measured by relating mean RT in the crossmodal condition to that in the unimodal conditions. The following definition quantifies the percent RT enhancement (Diederich and Colonius, 2004). For visual, auditory, and visual-auditory stimuli with expected reaction times E[RT_*V*_], E[RT_*A*_], and E[RT_VA_], respectively, *crossmodal response enhancement* (CRE) is defined as

(7) $$\text{CRE}=\{\begin{array}{ll} \frac{\min(\text{E}[RT_{V}],\text{ E}[RT_{A}])-\text{E}[RT_{VA}]}{\min(\text{E}[RT_{V}],\text{ E}[RT_{A}])}\;\cdot \;100, & \text{for RTP},\\ & \text{and}\\ \frac{\text{E}[RT_{V}]-\text{E}[RT_{VA}]}{\text{E}[RT_{V}]}\;\cdot \;100, & \text{for FAP,} \end{array}$$

where the visual is again taken as target modality in the FAP case. Replacing the means by the corresponding expressions from the TWIN model Equation (5) results in

(8) $$\text{CRE}=\{\begin{array}{ll} \frac{\min(\text{E}[V],\text{ E}[A])-\text{E}[\min(V,\;A)]+P(I_{\text{CSP}})\cdot \Delta }{\min(\text{E}[V],\text{ E}[A])+\mu }\;\cdot \;100, & \text{for CSP},\\ & \text{and}\\ \frac{P(I_{\text{FAP}})\;\cdot \;\Delta }{\text{E}[V]+\mu }\;\cdot \;100, & \text{for FAP.} \end{array}$$

Assuming further that visual and auditory intensity are matched, such that E[*A*]=E[*V*], yields identical denominators in the above ratios. Comparing the corresponding numerators then reveals that response enhancement for CSP is at least as large as that for FAP because (1) *P*(*I*_FAP_) ≤ *P*(*I*_RTP_) and (2) the term min(E[*V*], E[*A*]) − E[min(*V*, *A*)], the amount of statistical facilitation, is always non-negative. Therefore, we have

(9) CRE(CSP)≥CRE(FAP).

This result holds if Δ > 0, in analogy to the result derived above for crossmodal expected reaction time. Note that it is possible to have an observed CRE(CSP) of zero even if Δ is different from zero: it may have a negative amount just outweighing the statistical facilitation effect.

### 3.3. The effect of intensity variation on crossmodal response enhancement

According to the TWIN model assumptions, a direct effect of stimulus intensity only occurs in the peripheral processing channels. In later processing stages, direction and amount of crossmodal interaction are assumed to be modulated by intensity only via the outcome of first-stage processing, i.e., whether or not integration takes place. Obviously, any intensity variation that increases the likelihood that the peripheral processes terminate within a time window will lead to an increase in the crossmodal effect. This prediction has found ample empirical support. For example, in CSP the largest RT facilitation is typically found when stimulus intensities for both modalities are matched (“physiological synchronicity”; e.g., Corneil et al., 2002). In FAP, intensity effects become a bit more complex: first, increasing the intensity of a relatively weak visual target stimulus will speed up visual peripheral processing up to some minimum level, thereby increasing the chance for the visual target to win the race. Thus, the probability that the window of integration opens decreases, predicting less crossmodal interaction. Increasing the intensity of a non-target auditory stimulus, on the other hand, leads to the opposite prediction: the auditory stimulus will have a better chance to win the race and to open the window of integration, hence predicting more crossmodal interaction, on average. If SOA is varied as well, further distinctions can be made that will not be considered here.

### 3.4. The emergence of inverse effectiveness

In order to further examine the effect of intensity variation on CRE in the TWIN model, we introduce some distributional assumptions for the first stage processing times. These peripheral processing times, *V* for the visual and *A* for the visual stimulus, are assumed to have exponential probability distributions with positive-valued parameters λ_*V*_ and λ_*A*_, respectively. That is,

$$\begin{array}{l} f_{V}(t)=\lambda_{V}\exp[-\lambda_{V}t],\\ f_{A}(t)=\lambda_{A}\exp[-\lambda_{A}t] \end{array}$$

for *t* ≥ 0, and *f*_*V*_(*t*) = *f*_*A*_(*t*) ≡ 0 for *t* < 0. The exponential assumption is primarily motivated by its mathematical simplicity. Together with a Gaussian distribution assumption for second stage processing time^3^ the resulting distribution is a mixture of ex-Gaussian distributions for total reaction time, which has been demonstrated to be a reasonably adequate description for many empirically observed reaction time data (cf. Van Zandt, 2002).

For the probability of integration in FAP, we get

$$\begin{array}{l} \text{Pr}(I_{\text{FAP}})=\text{Pr}(A<V<A+\omega )\\ \;=\int_{0}^{\infty }f_{A}(t)[F_{V}(t+\omega )-F_{V}(t)]\;dt\\ \;=\int_{0}^{\infty }\lambda_{A}\exp[-\lambda_{A}t]\{\exp[-\lambda_{V}t]-\exp[-\lambda_{V}(t+\omega )]\;dt\}\\ \;=\frac{\lambda_{A}}{\lambda_{A}+\lambda_{V}}\{1-\exp[-\lambda_{V}\omega ]\}. \end{array}$$

Similarly, for the probability of integration in CSP, we get

$$\begin{array}{l} \text{Pr}(I_{\text{CSP}})=\text{Pr}(A<V<A+\omega )+\text{Pr}(V<A<V+\omega )\\ \;=\frac{\lambda_{A}}{\lambda_{A}+\lambda_{V}}\{1-\exp[-\lambda_{V}\omega ]+\frac{\;\lambda_{V}}{\lambda_{A}+\lambda_{A}}\{1-\exp[-\lambda_{A}\omega ] \end{array}$$

Assuming matching intensity levels again (that is, λ_*V*_=λ_*A*_≡λ) this simplifies to

(10) $$\text{Pr}(I_{\text{CSP}})=1-\exp[-\lambda \omega ]\equiv 2\text{ Pr}(I_{\text{FAP}}).$$

It is now straightforward to compute the crossmodal response enhancement expressions,

(11) $$\text{CRE}=\{\begin{array}{ll} \frac{{\left(2\lambda \right)}^{-1}+(1-e^{-\lambda \omega })\;\cdot \;\Delta }{\lambda^{-1}+\mu }\;\cdot \;100, & \text{for CSP},\\ & \text{and}\\ \frac{(1-e^{-\lambda \omega })\;\cdot \;\Delta }{2(\lambda^{-1}+\mu )}\;\cdot \;100, & \text{for FAP.} \end{array}$$

Inspection of these expressions reveals that crossmodal response enhancement, for both CSP and FAP, increases as a function of the facilitation parameter (Δ > 0) and the window width (ω), but decreases as a function of second stage processing time without crossmodal interaction (μ), as one would expect.

Intriguingly, the effect of increasing intensity parameter λ is different for the two paradigms: For FAP, CRE *increases* with λ (for Δ>0) no matter the values of the remaining parameters. Note that this is no contradiction to the observations in the previous section since here we are assuming identical λ parameters for target and non-target.

**Figure 1:**
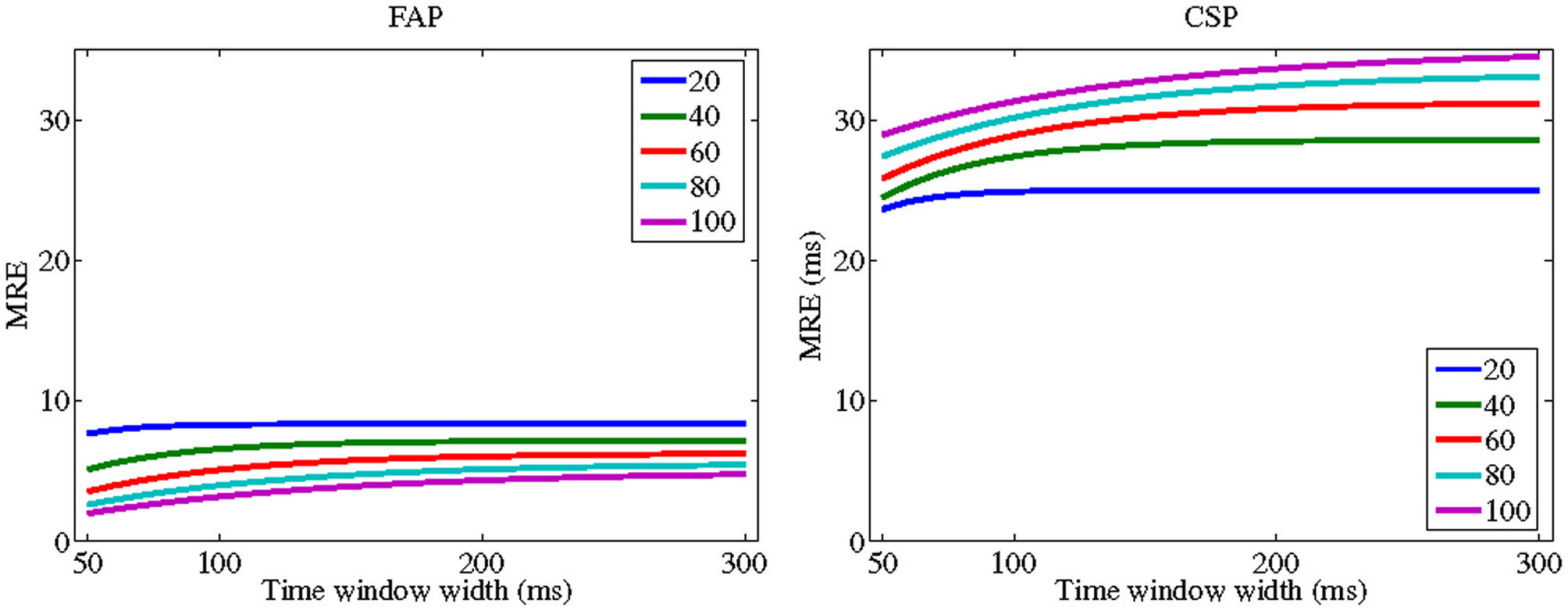
**TWIN predictions for crossmodal response enhancement (CRE) for focused attention paradigm (FAP) (left panel) and crossmodal signals paradigm (CSP) (right panel) as a function of time window width (ω)**. Each curve corresponds to a specific intensity parameter of the stimuli demonstrating a “inverse effectiveness” for CSP. The peripheral processing times for the auditory and visual stimuli are 1/λ_*A*_ = 1/λ_*V*_ equal to 20 (blue line); 40 (green); 60 (red); 80 (cyan); and 100 (magenta). Mean second stage processing time is μ = 100. Interaction parameter is Δ = 20.[all values in ms].

For CSP, however, CRE *decreases* with λ for many plausible values of the other parameters. Thus, TWIN's prediction here concurs with the “principle of inverse effectiveness” according to which crossmodal facilitation is strongest when stimulus strengths are weak or close to threshold level (Meredith and Stein, 1986). Figure 1 illustrates this finding for specific parameters and shows that it holds across all values of window width. Note that the difference between FAP and CSP with respect to “inverse effectiveness” is mainly due to an additional term in the numerator of the CRE equation (Equation 11) for CSP. This term, $\frac{1}{2\lambda }$, is the amount of statistical facilitation, min(E[*V*], E[*A*]) − E[min(*V*, *A*)]. Thus, here the “principle of inverse effectiveness” is based on the fact that statistical facilitation becomes the smaller the higher the intensity levels of the stimuli are. This observation suggests that, at least in the domain of reaction time measurement, “inverse effectiveness” may in part be a purely statistical effect. Because this result has been derived under the auxiliary assumption of exponentially distributed peripheral processing durations and is limited to certain, though plausible, parameter combinations, its remains to be shown whether it can be generalized to a larger class of distributions.

## 4. Optimal time windows for FAP and CSP

The effect of adding information from another modality should be particularly strong in an adverse environment, i.e., with a low signal-to-noise ratio (SNR). The *prima facie* plausibility of the inverse effectiveness principle is actually based on this idea. Within the TWIN framework, this would correspond to adjusting the size of the time window with respect to SNR, i.e., widening it for lower SNR values. Note that this differs from the above discussion of the effect of stimulus intensity where time window size was assumed to be constant across trials. The perspective taken now is that the adjustment of the time window is a *top-down process* occurring only if there are long-term changes in the environment as measured by SNR or, possibly, as a consequence of changes in the cost/benefit of integration. This raises the question of how an appropriate window size should be determined.

Clearly, an infinitely large time window would lead to mandatory integration, and one could argue that this is what, e.g., a sufficiently low SNR would require. A more elaborate response, however, is based on the hypothesis that integrating crossmodal information always involves a possibly implicit decision about whether or not two (or more) sensory cues originate from the same event, i.e., have a common cause and that integration should only occur in that case (e.g., Stein and Meredith, 1993; Koerding et al., 2007). For example, in a predator-prey situation it may be vital for the potential prey to recognize whether a sudden movement in the dark is caused by a predator or a harmless wind gust. If visual information is accompanied by some vocalization from a similar direction, it may be adequate to respond to the potential threat by assuming that the visual and auditory information are caused by the same source, i.e., to perform multisensory integration leading to a speeded escape reaction. On the other hand, in a rich dynamic environment it may also be disadvantageous, e.g., leading to a depletion of resources, or even hazardous, to routinely combine information associated with sensory events which—in reality—may be entirely independent and unrelated.

Colonius and Diederich (2010) introduced a decision-theoretic approach for finding an optimal time window that is in line with this setup. Subsequently, we have derived an explicit expression for the optimal time window for the FAP case (Colonius and Diederich, 2011). Here, we present an optimal time window for CSP as well and discuss how predictions for MRE under optimal performance differ between the two paradigms. To keep this paper self-sustained, the next two sections summarize our previously obtained results.

### 4.1. Basic decision situation and optimal decision rule

The basic decision situation is presented in a schematic manner by the following *payoff matrix* (Table 1). It defines the gain (blue) or cost (red) function *U* associated with the *states of nature* (*C*) and the *action* (*I*) of audiovisual integration: Variable *C* indicates whether visual and auditory stimulus information are generated by a common source (*C*=1), i.e., an *audiovisual event*, or by two separate sources (*C*=2), i.e., auditory and visual stimuli are unrelated to each other. Variable *I* indicates whether or not integration occurs (*I*=1 or *I*=0, respectively). The values *U*_11_ and *U*_20_ correspond to correct decisions and will in general be assumed to be positive numbers, while *U*_21_ and *U*_10_, corresponding to incorrect decisions, will be negative. The organism's task is to balance these costs and benefits of multisensory integration by an appropriate optimizing strategy.

**Table 1:** **Payoff matrix for the basic decision situation**.

**Gain/Cost**	**Integration (*I*=1)**	**No integration (*I*=0)**
Common source (*C*=1)	*U*_11_	*U*_10_
Separate sources (*C*=2)	*U*_21_	*U*_20_

In order to derive an optimal decision rule, we assume that *a-priori* probabilities for the events {*C* = i}_i = 1, 2_ exist, with Pr(*C* = 1) = 1 − Pr(*C* = 2). In general, an optimal strategy may involve many different aspects of the empirical situation, like spatial and temporal contiguity. As a simplifying starting point, the temporal disparity between the “arrival times” of the unimodal signals is assumed to be the *only* perceptual evidence utilized by the organism. Thus, computation of an optimal time window will be based on the prior probability of a common cause and the likelihood of temporal disparities between the unimodal signals; that is, we define the *likelihood function* *f*(*t*| *C*), where *f* denotes the probability mass function or, if it exists, the density function of *T* given *C* takes on a value. Using Bayes' rule, we immediately have the *posterior* probability of a common cause given the occurrence of an arrival time difference *t*,

$$\text{Pr}(C=1|t)=\frac{f(t|C=1)\text{Pr}(C=1)}{f(t|C=1)\text{Pr}(C=1)+f(t|C=2)\text{Pr}(C=2)}.$$

On each trial, in order to maximize the expected value E[*U*] of function *U* in the payoff matrix (Table 1), the decision-maker is to choose that action alternative (i.e., to integrate or not) which contributes, on the average, more to E[*U*] than the other action alternative. Introducing the *likelihood ratio* function

L(t)=f(t|C=1)/f(t|C=2),

results in the following decision rule (cf. Colonius and Diederich, 2010):

(12) $$“If\;L(t)>\frac{\text{Pr}(C=2)}{\text{Pr}(C=1)}\times \frac{U_{2\;0}-U_{2\;1}}{U_{1\;1}-U_{1\;0}},\;integrate,\;otherwise\;do\;not\;integrate.”$$

This decision rule implicitly defines a window that is optimal in the sense of maximizing E[*U*]:

*The* optimal time window *is the set of all values of absolute arrival time differences* {*T* = *t*} *satisfying the inequality in the above decision rule (12)*.

The effect of the prior probability for a common cause on the time window is immediately predictable from this decision rule: Keeping the *U*-values constant, the expression on the right of inequality (12) will decrease as *P*(*C* = 1) increases, implying an extension of the time window.

### 4.2. Computing an optimal time window for FAP

In order to compute the optimal time window, we must specify the likelihood function. For two separate sources we assume a uniform law,

(13) $$f(t|C=2)=\{\begin{array}{ll} 1/(t_{1}-t_{0}) & \text{if }t_{\text{0}}<t<t_{\text{1}},\\ 0 & \text{otherwise,} \end{array}.$$

Here, *t*_0_, *t*_1_ are real numbers defining the *observation interval*, that is, the interval of time limiting all possible ATDs due to the construction of the trial length by the experimenter. Thus, under two separate sources any arrival time difference is assumed to occur with the same likelihood within the observation interval (*t*_0_, *t*_1_).

For a single source, we postulate^4^ that the likelihood function is induced by the distribution of the peripheral processing times *V* and *A*. For the FAP, given the independent exponential distribution assumption for *V* and *A* in TWIN, the distribution of arrival time differences under a common source, *V*−A, can be shown to be an *asymmetric Laplace distribution* (Colonius and Diederich, 2011):

(14) $$f(t|C=1)=\frac{\lambda_{V}\lambda_{A}}{\lambda_{V}+\lambda_{A}}\times \{\begin{array}{ll} \exp(-\lambda_{V}t) & \text{if }t\geq \text{0},\\ \exp(\;\lambda_{A}t) & \text{if }t<0. \end{array}$$

Note that the asymmetry derives from the asymmetry of the role of the modalities in FA tasks (target vs. non-target). For *t*_0_ ≤ *t* ≤ *t*_1_, the likelihood ratio becomes^5^

(15) $$L(t)\;=\;f(t|C=1)/f(t|C=2)=\frac{\lambda_{V}\lambda_{A}}{\lambda_{V}+\lambda_{A}}(t_{1}-t_{0})\times \{\begin{array}{ll} \exp(-\lambda_{V}t) & \text{if }t\in (t_{\text{0}},\;t_{\text{1}})\cap [\text{0},\;t_{\text{1}}),\\ \exp(\lambda_{A}t) & \text{if }t\in (t_{\text{0}},\;t_{\text{1}})\cap (t_{\text{0}},\text{ 0}] \end{array}$$

To simplify the exposition, in the following the ratio of utility differences occurring in Equation 12 will be set equal to one. Thus, according to the optimal decision rule, audiovisual integration should be performed if and only if

$$L(t)>\frac{1-p}{p},$$

with *p* ≡ Pr(*C* = 1). Assuming matching intensity levels (λ ≡ λ_*A*_ = λ_*V*_), inserting the expression for *L*(*t*) from Equation 15, and solving for *t* yields the following *optimal* time window for *t* ∈ (*t*_0_, *t*_1_):

(16) $$\left\{t|\frac{1}{\lambda }\log\left[\frac{2(1-p)}{\lambda (t_{1}-t_{0})p}\right]\leq t\leq \frac{1}{\lambda }\log\left[\frac{\lambda (t_{1}-t_{0})p}{2(1-p)}\right]\right\}$$

provided that

(17) $$\frac{2(1-p)}{\lambda (t_{1}-t_{0})p}\leq 1.$$

This latter condition guarantees that the left side of the interval is non-positive and the right side is non-negative. For the width of the optimal time window, we get immediately

(18) $$\omega_{\text{opt}}=\left(\frac{2}{\lambda }\right)\log\left[\frac{\lambda (t_{1}-t_{0})}{2}\frac{p}{1-p}\right]$$

This is obviously an increasing function of the prior odds *p*/(1 − *p*) and of the observation interval (*t*_0_, *t*_1_). Increasing *P*(*C* = 1) leads to a widening of the time window, in this case approaching infinity in a non-linear fashion. Moreover, the optimal time window disappears for values of the prior below a certain positive threshold value. Although the exact threshold value depends on the experimental context (i.e., *t*_1_− *t*_0_ and λ) and may get close to zero, this prediction provides a potentially strong model test: for a small enough value of *P*(*C* = 1) there should be no multisensory integration effect at all.

### 4.3. Computing an optimal time window for CSP

The derivation of an optimal time window for CSP is analogous to the FAP case, except that now the likelihood is defined using the *absolute* arrival time difference of the unimodal signals, *T*=|*V* − *A*|. Given the assumption of independent exponential distribution for *V* and *A* in TWIN, the distribution of *T* under a common source, then turns out to be a mixture of exponential distributions:

$$\begin{array}{l} \text{Pr}(|V-A|\;\leq t)\;=\text{Pr}(V<A<V+t)+\text{Pr}(A<V<A+t)\\ \;F_{T}(t|C=1)\;=\int_{0}^{\infty }f_{V}(v)[F_{A}(v+t)-F_{A}(v)]dv+\int_{0}^{\infty }f_{A}(a)[F_{V}(a+t)-F_{V}(a)]da\\ \;=\frac{\lambda_{V}}{\lambda_{A}+\;\lambda_{V}}\left\{1-\exp[-\lambda_{A}t]\right\}+\frac{\lambda_{A}}{\lambda_{A}+\;\lambda_{V}}\left\{1-\exp[-\lambda_{V}t]\right\}. \end{array}$$

Differentiation then yields the density for |*V* − *A*|:

(19) $$f_{T}(t|C=1)=\frac{\lambda_{V}\lambda_{A}}{\lambda_{A}+\;\lambda_{V}}\left\{\exp[-\lambda_{A}t]+\exp[-\lambda_{V}t]\right\},$$

from which the likelihood ratio follows:

(20) $$L(t)=(t_{1}-t_{0})f_{T}(t)$$

which is defined for *t* ∈ (*t*_0_, *t*_1_). It is easy to see that *L*(*t*) is monotonically decreasing in *t*; thus, larger arrival time differences, positive or negative, provide evidence in favor of two separate sources rather than a single source, as is to be expected.

Inserting the expression for *L*(*t*) from Equation 20 and solving for *t* yields the following *optimal* time window with λ ≡ λ_*A*_ = λ_*V*_:

(21) $$\left\{t|0\leq t<\frac{1}{\lambda }\log\left[\frac{p}{1-p}\lambda (t_{1}-t_{0})\right]\right\}$$

for *t* ∈ (*t*_0_, *t*_1_). In order to exclude negative values of the logarithm,

$$p\geq {\left[\lambda (t_{1}-t_{0})+1\right]}^{-1}$$

must hold. The upper bound of the optimal time window is identical to its length. As in FAP, it is obviously an increasing function of the prior odds *p*/(1 − *p*) and of the observation interval (*t*_0_, *t*_1_). Increasing *p* = P(*C* = 1) leads to a widening of the time window, approaching infinity in a non-linear fashion. Moreover, as before, the optimal time window disappears for values of the prior below a certain positive threshold value, providing a potential model test since for a small enough value of *P*(*C* = 1) there should be no multisensory integration effect at all.

### 4.4. CSP vs. FAP: comparing optimal time window width and CRE

**Figure 2:**
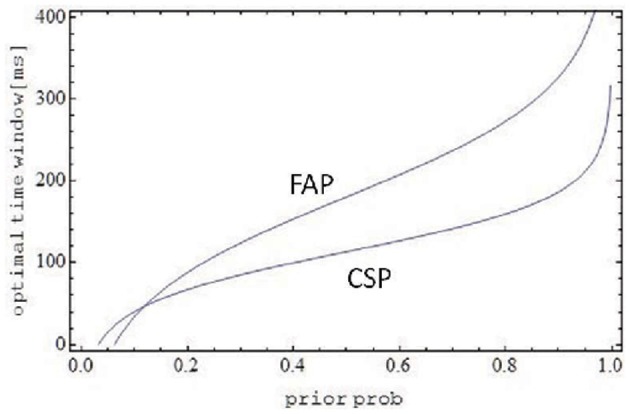
**Optimal time window as a function of prior probability *p* for a common source**. Except for very small *p*, the optimal window size for FAP is larger than for CSP, compensating for the lower probability of integration in FAP compared to CSP. Parameters are λ = 0.03, *s* = 1 s.

We are now in a position to compare both paradigms with respect to their optimal time window width and the magnitude of their multisensory response enhancement under optimality. For the optimal time window size, ω_opt_,

(22) $$\omega_{\text{opt}}=\{\begin{array}{ll} \frac{2}{\lambda }\log\left[\frac{\lambda }{2}\frac{p}{1-p}s\right] & \text{for FAP;}\\ \frac{1}{\lambda }\log\left[\lambda \frac{p}{1-p}s\right] & \text{for CSP} \end{array}$$

under the provision that the logarithmic term does not become negative. Note that the length of the observation interval (*s* ≡ *t*_1_ − *t*_0_), being determined by the experimental setup, can be considered an inessential scaling factor. Not surprisingly, as observed before, both optimal window widths increase with increasing prior *p* for a common cause, approaching infinity for *p* → 1. Figure 2 shows optimal time window width for both FAP and CSP as a function of the prior *p*. The width for FAP is larger than for CSP nearly everywhere, except for rather small values (depending on the scaling factor *s*) of the prior, where the opposite holds. This make sense intuitively: the probability of integration in FAP is only half the size of the probability of integration in CSP (cf. Equation 10). Thus, for a fixed and not too small prior, window size in FAP must increase in order to match the probabilities of integration in both paradigms^6^. Inspection of ω_opt_ (Equation 22) reveals that the effect of intensity parameter λ is more complex. For small values of *p* it is non-monotonic (increasing, then decreasing) and for larger *p* values ω_opt_ it decreases for both FAP and CSP. The latter observation may reflect a moderating effect of intensity on window size once the window already is rather large.

Inserting ω_opt_ into the expressions for crossmodal response enhancement (CRE) yields

(23) $$\text{CREopt}=\left\{ \begin{array}{l} \begin{array}{cc} {\left(1/\lambda +\mu \right)}^{-1}\left[\frac{1}{2\lambda }+\Delta \left(1-\frac{1-p}{\lambda ps}\right)\right]\times 100 & \text{for CSP}, \end{array}\\ \begin{array}{cc} & \text{ and} \end{array}\\ \begin{array}{cc} {\left(1/\lambda +\mu \right)}^{-1}\Delta /2\left[1-{\left(\frac{2(1-p)}{\lambda ps}\right)}^{2}\right]\times 100, & \text{for FAP.} \end{array} \end{array}\right.$$

We know from Ineq. 9 that CRE(FAP) cannot be larger than CRE(CSP) when the parameters ω, λ, Δ (Δ > 0), and μ are all identical for the two paradigms. However, since now the optimal window widths are not identical for CSP and FAP, this ordering might no longer hold. Closer scrutiny of the above equations reveals, however, that crossmodal response enhancement in CSP still dominates the one in FAP when the other parameters are kept the same. Moreover, for λ increasing without bound, CRE(CSP) will become twice as large as CRE(FAP).

## 5. Summary and conclusion

Assuming exponential arrival time distributions, the framework of the TWIN model has been specified here so that specific quantitative predictions could be made comparing the FAP and the CSP with respect to (1) the probability of multisensory integration and (2) expected crossmodal response enhancement (reaction time facilitation/inhibition). Moreover, introducing a decision-theoretic framework for TWIN, the investigation could be extended to comparing the CSP and FAP paradigms with respect to their predicted optimal time windows. Glossing over some of the required conditions concerning the specific parameter values, the main findings were:

– the probability of crossmodal integration for CSP is twice the probability of integration for FAP;
– crossmodal response enhancement (facilitation) for CSP is at least as large as for FAP;
– TWIN model is consistent with the occurrence of a “inverse effectiveness” under the CSP but not under FAP;
– within the decision-making framework for TWIN, explicit expressions for the computation of time windows of *optimal* width for both CSP and FAP have been derived;
– the optimal time window is larger for FAP than for CSP across (nearly) all values of the prior probability (of a common source for both modalities), thereby compensating for the smaller probability of integration in FAP (see first item on this list)
– optimal crossmodal response enhancement (facilitation) for CSP is larger than for FAP (or at least as large) even though their optimal window widths differ.

The obvious next step will be to test these predictions experimentally. Apart from a pilot study in our lab (cf. Colonius and Diederich, 2012), we are not aware of any systematic empirical studies comparing FAP and CSP under matching stimulus intensity levels. In particular, studies are needed varying the prior probability of a common source in order to test the above predictions concerning optimality (for FAP, see Van Wanrooij et al., 2010). An unsolved issue, for example, is whether data that are not consistent with optimality indicate sub-optimal behavior or are simply due to participants' subjective priors deviating from the objective priors. Moreover, except for the first two items in the above list, the current predictions have been derived under the hypothesis of independent exponential arrival time distributions. It remains to probe by further analysis whether or not these predictions can be generalized to other plausible distributions, e.g., gamma distributions.

A fundamental difference between the tasks in FAP and CSP is that in the focused attention paradigm there must be a mechanism to distinguish a target- from a non-target-modality stimulus at a very early stage of processing, whereas in the CSP such a mechanism is not required. This difference between paradigms is in line with a recent suggestion in Kayser et al. (2010) of two different modes of multisensory integration, one occurring in a detection task where the response to weak stimuli is enhanced, and another occurring in discrimination and identification tasks where the precision and reliability of the responses are improved (see also the commentary by Ghazanfar and Lemus, 2010). This, in turn, suggests to probe whether, in focussed attention data, one effect of the non-target-modality stimulus is to diminish the variability of crossmodal reaction times, relative to the unimodal variability. In the TWIN model, no explicit mechanism to distinguish target- from non-target modalities has been implemented yet, but this may be called for if one attempts to investigate such hypotheses.

Given that the TWIN model predicts changes in the (optimal) time window as a function of the prior probability of a common source, the basic question about the malleability of the time window arises. There are a number of recent studies, using other experimental paradigms, that provide evidence for a dynamic adaptation of the time window to changes in context. For example, using a simultaneity judgment task, Powers and colleagues showed that significant and lasting changes of perceived simultaneity (40% narrowing in the width of the window) can be induced after a single day of training (Powers et al., 2009) and are accompanied by decreases in BOLD activity within a network of multisensory and unisensory areas (Powers et al., 2012)^7^. Nevertheless, direct evidence in the context of the reaction time paradigm will only be provided by the type of experimental tests suggested above.

### Conflict of interest statement

The authors declare that the research was conducted in the absence of any commercial or financial relationships that could be construed as a potential conflict of interest.

## Footnotes

^1^The terms “redundant targets” or “redundant signals” paradigm are more common but do not explicitly refer to stimuli coming from different sensory modalities.

^2^For simplicity, we are using *V* and *A* for the crossmodal condition in the remainder of this paper, although this could be replaced by any other pair of modalities. Moreover, without losing much generality—since non-zero SOA values can be subsumed as additive constants under *V* or *A*—we suppress any reference to values of SOA different from zero.

^3^That is, a convolution of an exponential and a Gaussian distribution.; for an alternative, replacing the Gaussian by the Wald distribution, see Schwarz, 2001.

^4^It is important to keep in mind that this is an additional assumption not directly following from the decision framework. It seems plausible, however, given that in a typical environment visual and auditory information deriving from a common source should occur more or less at the same point in time (cf. Leone and McCourt, 2012).

^5^Note that for *t* outside of the observation interval the likelihood ratio remains undefined.

^6^All other conditions being equal there is no *a-priori* reason why the optimal *probability* of integration should differ between FAP and CSP.

^7^For a recent review of the general area of perception of synchrony, see Keetels and Vroomen (2012).
